# Effect of pre-germinated brown rice intake on diabetic neuropathy in streptozotocin-induced diabetic rats

**DOI:** 10.1186/1743-7075-4-25

**Published:** 2007-11-23

**Authors:** Seigo Usuki, Yukihiko Ito, Keiko Morikawa, Mitsuo Kise, Toshio Ariga, Michael Rivner, Robert K Yu

**Affiliations:** 1 Institute of Molecular Medicine and Genetics, Medical College of Georgia, Augusta, GA 30912, USA; 2 Department of Neurology, Medical College of Georgia, Augusta, GA 30912, USA; 3 FANCL Research Institute, FANCL Corporation, Yokohama, 244-0806, Japan

## Abstract

**Background:**

To study the effects of a pre-germinated brown rice diet (PR) on diabetic neuropathy in streptozotocin (STZ)-induced diabetic rats.

**Methods:**

The effects of a PR diet on diabetic neuropathy in STZ-induced diabetic rats were evaluated and compared with those fed brown rice (BR) or white rice (WR) diets with respect to the following parameters: blood-glucose level, motor-nerve conduction velocity (NCV), sciatic-nerve Na^+^/K^+^-ATPase activity, and serum homocysteine-thiolactonase (HTase) activity.

**Results:**

Compared with diabetic rats fed BR or WR diets, those fed a PR diet demonstrated significantly lower blood-glucose levels (*p *< 0.001), improved NCV (1.2- and 1.3-fold higher, respectively), and increased Na^+^/K^+^-ATPase activity (1.6- and 1.7-fold higher, respectively). The PR diet was also able to normalize decreased serum homocysteine levels normally seen in diabetic rats. The increased Na^+^/K^+^-ATPase activity observed in rats fed PR diets was associated with elevations in HTase activity (r = 0.913, *p *< 0.001). The *in vitro *effect of the total lipid extract from PR bran (TLp) on the Na^+^/K^+^-ATPase and HTase activity was also examined. Incubation of homocysteine thiolactone (HT) with low-density lipoprotein (LDL) *in vitro *resulted in generation of HT-modified LDL, which possessed high potency to inhibit Na^+^/K^+^-ATPase activity in the sciatic nerve membrane. The inhibitory effect of HT-modified LDL on Na^+^/K^+^-ATPase activity disappeared when TLp was added to the incubation mixture. Furthermore, TLp directly activated the HTase associated with high-density lipoprotein (HDL).

**Conclusion:**

PR treatment shows efficacy for protecting diabetic deterioration and for improving physiological parameters of diabetic neuropathy in rats, as compared with a BR or WR diet. This effect may be induced by a mechanism whereby PR intake mitigates diabetic neuropathy by one or more factors in the total lipid fraction. The active lipid fraction is able to protect the Na^+^/K^+^-ATPase of the sciatic-nerve membrane from the toxicity of HT-modified LDL and to directly activate the HTase of HDL.

## Background

Pre-germinated brown rice (PR), prepared by soaking brown rice (BR) in 37°C water for 24 h to initiate germination of sprouts not exceeding 0.5-mm in length [1], is a commercial dietary supplement in Japan. The efficacy of PR-enriched diets in reducing blood glucose levels in streptozotocin (STZ)-induced diabetic rats has been reported [2]. STZ, which induces type 1 diabetes mellitus when delivered to rats, is a useful etiological model for studying complications caused by diabetic hyperglycemia.

In clinical studies, we previously showed that both non-diabetic patients and hyperglycemic patients with uncontrolled diabetes who ate diets supplemented with PR had lower postprandial blood-glucose levels than those who ate white rice (WR)-supplemented diets [3,4]. It has been suggested that the reduction in blood- glucose levels and the incidence of diabetic vascular complications in diabetic patients fed a PR-rich diet may result from the substantially higher dietary fiber content of germinating rice bran [5]. However, the γ-aminobutyric acid (GABA) produced during germination of BR or the vitamins, minerals and/or unknown bioactive lipids in the bran and germ layer of PR may also be associated with PR's effect on blood-glucose levels [6,7]. Reduced type-1 plasminogen-activator inhibitor and lipid-peroxide levels have been reported in diabetic rats [2], suggesting that PR intake may ameliorate diabetic vascular complications such as retinopathy and nephropathy.

Another major complication of diabetes is peripheral neuropathy, which occurs in greater than 50% of diabetics. Early nervous system dysfunctions in diabetics include reduced nerve conduction velocity (NCV), which frequently is followed by axonal degeneration and paranodal demyelination [8]. It is of interest, therefore, to compare PR and BR diets during the development of diabetic neuropathy. We undertook a study to investigate the difference in the effectiveness of PR and BR diets in rats with STZ-induced diabetic neuropathy.

The neuropathy seen in the well-characterized STZ-induced diabetic rat model shares many of the same pathological, functional, and biochemical alterations seen in human diabetic [9,10]. In particular, gradual decreases in Na^+^/K^+^-ATPase activity and NCV are observed in the sciatic nerves of STZ-treated rats. Recent findings suggest that depressed activity of Na^+^/K^+^-ATPase, a plasma membrane-bound enzyme, is a hallmark of diabetic neuropathy [11]. PR-enriched diets have shown promise in ameliorating many vascular diabetic sequelae in human patients according to Japanese folk medicine; however, there is little systematic scientific study regarding its nutritional functions. We were interested in investigating the comparative effects of PR- and BR-enriched diets on the development of diabetic neuropathy in STZ-induced diabetes in rats. We decided to investigate the effect of PR- and BR-enriched diets specifically on NCV and sciatic nerve Na^+^/K^+^-ATPase activities in STZ-induced diabetes in rats.

Diabetic neuropathy is also thought to develop as a result of damage caused by oxidative stress and other vascular risk factors [12,13]. Homocysteine-thiolactone (HT) is formed from an excess amount of serum homocysteine (Hcy) and causes protein homocysteinylation and subsequent protein damage. In particular, homocysteinylated low-density serum lipoproteins (LDLs) are more susceptible to lipid peroxidation. On the other hand, high-density lipoproteins (HDLs) are resistant to homocysteinylation because of an associated homocysteine-thiolactonase (HTase) that hydrolyzes HT; this hydrolysis prevents subsequent HDL lipid peroxidation. HT is reported to produce oxidative stress in rat hippocampal neurons by inhibiting Na^+^/K^+^-ATPase activity [14,15]. Further, Vignini et al. [16] reported that HT-modified LDL attenuates Na^+^/K^+^-ATPase activity in cultured human aortic endothelial cells.

HT is thought to be an endogenous substrate for three isozymes of the serum esterase paraoxonase (PON): PON1, PON2, and PON3 [17]. PON1 has been shown to be involved in the detoxification of various organophosphatases, such as nerve gases, dietary neurotoxins, or toxic lipids produced during oxidative stress [18]. Serum PON1 activity is decreased in type 2 diabetic patients with atherosclerosis [19] and in STZ-induced type 1 diabetic rats [20]. Decreased PON1 activity is also observed in patients with type 1 and type 2 diabetic peripheral neuropathy, suggesting that diabetic neuropathy may also arise, in part, due to the increased susceptibility of the nervous system to neurotoxic damage resulting from the lack of protection in diabetics normally afforded by PON1 in non-diabetics [21]. The role of PON1 activity in HDL in diabetes has become an important concern [22,23]. For this reason, HTase activity can be another important biomarker for evaluating diabetic neuropathy, especially when estimation of this enzyme activity requires HT to be a natural substrate. To assess the effects of a PR-enriched diet on the development of diabetic neuropathy, we also evaluated the effect of PR- and BR-enriched diets on serum PON1 activities in STZ-induced diabetic rats.

In addition, circumstantial evidence suggests that other bioactive components are present in PR bran that are more potent than GABA in alleviating diabetic neuropathy. To test this possibility, we isolated the total lipid extracts (TLp) from PR and BR and compared the effects of these lipid fractions on HTase activity.

## Methods

### STZ-induced diabetic rats and experimental diets

Male Wistar rats, weighing 120–140 g, received intra-peritoneal injections of STZ (65 mg/kg in 100 mM sodium citrate buffer, pH 4.5). The use of these animas had been approved by Medical College of Georgia's Institutional Animal Care and Use Committee. Beginning 1 week after STZ injection, blood samples were obtained at each time point from the same rats following a 15-hour starvation period by pricking the tip of the rat tail and bleeding a drop (about 0.06 ml) of blood. The whole blood was obtained from each rat by heart puncture at the endpoint of the experiment. The blood-glucose levels were measured using a strip-operated blood glucose meter (Accu-Chek Advantage Blood Glucose Meter, Roche Diagnostics, Indianapolis, IN). Two animals were housed per cage in a controlled environment. All animals were allowed free access to water and food, except during the starvation periods just prior to blood glucose assessments.

All rice and control diets were manufactured as powdered feed by Harlan Teklad (Madison, WI). The control diet (AIN93G) was composed of cornstarch [39.7% (w/w)], α-cornstarch [13.2% (w/w)], casein [20.0% (w/w)], L-cysteine [0.3% (w/w)], sucrose [10% (w/w)], soybean oil [7.0% (w/w)], cellulose powder [5.0% (w/w)], mineral mix [3.5% (w/w)], vitamin mix [1.0% (w/w)], choline bicitrates [0.25% (w/w)], and_butylhydroquinone [0.0014% (w/w)]. Pre-germinated brown (PR), brown (BR), or white (WR) rice diets were produced by replacing cornstarch and α-cornstarch with pre-germinated brown, brown, or white rice, respectively (Table 1).

**Table 1:** Com position of experim ental diets (g/100 g)

Ingredient	AIN93G	WR	BR	PGR
Protein	17.7	21.5	21.6	21.8
Fat	7.2	7.0	8.3	8.1
Available carbohydrate	55.1	53.4	50.0	50.4
Dietary fiber	5.0	5.6	6.5	6.7
Total energy(kcal/100 g)	366.0	373.8	374.1	375.1

Values are calculated from ingredient analysis or m anufacturer data

### Animal experiment design

Male Wistar rats (n = 40) received intra-peritoneal injections of STZ and were maintained on AIN93G for 2 weeks. Only those animals whose blood glucose levels were > 400 mg/dl were considered diabetic. The diabetic rats (n = 31) were divided into four experimental groups: the DWR (n = 9), DBR(n = 9), and DPR (n = 9) groups received WR-, BR-, and PR-enriched diets, respectively, and DC group (n = 4) were fed AIN93G. Non-diabetic rats (n = 22) were also divided into four feeding groups: the WR (n = 6), BR (n = 6), and PR (n = 6)groups received WR-, BR-, and PR-enriched diets, respectively, while the C control group (n = 4) received AIN93G.

Because rats became moribund with severe diabetic deterioration initiating 3 weeks after STZ injection, the experimental endpoint was designated 3 weeks after initiation of the rice-enriched diet regimens (i.e., 5 weeks after STZ-injection). Food consumption was measured by weighing the feeding containers daily. Three weeks after initiation of the rice-enriched diets, tail-nerve conduction electrophysiological studies were performed, and then the rats were sacrificed. Whole serum samples were obtained for biochemical analyses as described below. The sciatic nerves were removed; the right sciatic nerve was utilized for morphometric studies while the left sciatic nerve was assayed for Na^+^/K^+^-ATPase activities.

### Nerve-conduction velocity measurements

Nerve-conduction velocities (NCVs, m/sec) were assessed in the rat tail nerve using a Nicolet VikingQuest EMG machine (Neurocaregroup, Madison, WI) according to the modified procedure of Anderson *et al*. [24]. In brief, the nerves were stimulated using external digital ring electrodes with twisted wires (Medtronic Functional Diagnostics, Skovlunde, Denmark) instead of needle electrodes [25]. The electrodes were placed in segments proximal (5 cm) and distal (2 cm) from the recording position (7 cm far from the joint of rat tail). Blood collection was from the tip of the tail that was distant and non-interactive with NCV measurement. NCV was evaluated from four different waves generated from electrical stimulations; each wave showed a reproducible pattern and the same amplitude level as the stimulator-voltage was increased. During each measurement, a constant surface temperature of the rat tail was maintained (34–35°C). NCV values represent an average value from 4 nerve conduction wave measurements per animal.

### Morphometric analysis

The right sciatic nerves were carefully dissected from their origin (5 mm distal to the gluteus maximus) through the distal branch point at the peroneal and tibial nerves in order to avoid stretching. These nerve sections were placed overnight in a fixative solution containing paraformaldehyde. After washing 3 times in cacodylate buffer (pH 7.2), the nerves were cross-sectioned into two pieces and embedded in epoxy resin (Poly/Bed 812, Polysciences Inc., Warrington, PA). Fascicle cross sections were stained with 1% toluidine blue and observed under an Axiophot photomicroscope equipped with an Axiocam (Carl Zeiss, Jena, Germany). Images were stored and analyzed using AxioVision. The total number of myelinated fibers in each fascicle was assessed by visual counting. In myelinated fibers, both the axonal and total fiber diameters were measured, based on the average of the major and minor diameters. Diameters of the myelinated fibers and axons, as well as the G ratio (axonal diameter/total fiber diameter, a measure of the degree of myelination), were obtained, and histograms of their distributions and the G ratios were constructed as percentages of size frequency.

### Total lipid preparation

The total lipid fractions from PR and BR bran (TLp and TLb, respectively) were prepared by extracting 5 g of bran twice with 30 ml and 20 ml of chloroform-methanol (1:1 (v/v) and 2:1 (v/v), respectively) using the procedure of Folch et al. [26]. Lipid fractions from these two extractions were combined, evaporated, and used in *in vitro *enzyme assays.

### Lipoprotein separation

Lipoprotein fractions were prepared from rat serum using previously published procedures [27]. Briefly, freshly collected serum obtained from normal male Wistar rats (n = 4) was pooled and adjusted to a density of 1.3 g/ml with solid potassium bromide. A discontinuous density gradient was formed in a centrifuge tube by layering normal saline (3.5 ml, 1.006 g/ml) over the adjusted serum (1.5 ml, 1.3 g/ml). Lipoproteins were separated by ultracentrifugation (370,000 × g, 45 min, 4°C) in a TV865 rotor. Three major lipoprotein fractions (VLDL, LDL, and HDL) were collected and dialyzed overnight against PBS at 4°C.

### Sciatic nerve membrane preparation for the Na^+^/K^+^-ATPase assay

Crude sciatic nerve membranes were prepared from each rat of the diet experiment using a previously published procedure [28]. Briefly, the left sciatic nerve from rats fed WR-, BR-, or PR-enriched diets, or AIN93G, or from untreated male Wistar rats were homogenized in Polytron homogenizer in a cold iso-osmotic solution containing 250 mM sucrose, 10 mM HEPES-Tris buffer (pH 7.6), EDTA 2 mM, and PSMF 1 mM. The homogenates were centrifuged for 10 min at 1,500 × g at 4°C; the supernatants were collected and then centrifuged at 191,000 × g for 45 min at 4°C. After decanting the supernatant, the pellets were resuspended in 100 μl of 250 mM sucrose in 10 mM HEPES-Tris buffer (pH 7.6).

### Na^+^/K^+^-ATPase assay

Na^+^/K^+^-ATPase activities were assayed as previously described [28]. Briefly, a 0.2 ml assay medium containing 10 mM MgCl_2_, 20 mM HEPES-Tris (pH 7.0), 120 mM NaCl, 30 mM KCl, 0.5 mg/ml of crude membrane preparations, and 25 mM [γ-^32^P]ATP (100,000 cpm) were incubated at 37°C for 15 min, followed by the addition of 0.1 ml of activated carbon (0.1 mg/ml). Parallel assays were also performed in which 1 mM ouabain was added to the assay medium. Following the addition of activated carbon, the samples were centrifuged at 1,500 × g for 15 min at 4°C, the supernatants were collected, and the cpm of the inorganic ^32^P radioactivity in the fractions was measured using a Beckman scintillation counter. Ouabain-sensitive Na^+^/K^+ ^ATPase activity was calculated by subtracting the ouabain-sensitive activity from the Na^+^/K^+^-enhanced activity.

### Paraoxonase 1 (PON1) activity assays

PON1 activity was measured in 96-well ELISA plates; each well contained 20 μl of rat serum (diluted 1:4 in Tris-HCl buffer) and 200 μl of substrate solution containing 5.5 mM paraoxoethyl and 2 mM CaCl_2 _in 100 mM Tris-HCl buffer (pH 8.0). Generation of *p*-nitrophenol was monitored as a function of time at 25°C by recording the absorbance at 412 nm using a spectrophotometer. Enzyme activities were calculated using the molar absorption coefficient of *p*-nitrophenol (ε_412_^M^, 169,000 M^-1 ^cm^-1^).

### Homocysteine-thiolactonase (HTase) activity assays

HTase activities in rat serum or in HDL fractions from normal male Wistar rats were measured with a commercial assay kit (Alfresa Auto HTLase; Alfresa Pharma Corp., Osaka, Japan) [23] that utilizes γ-thiobutyrolactone as the substrate. HTase hydrolyzes the lactone ring, generating free thiol, which reacts with 5, 5'-dithio-bis (2-nitrobenzoic acid). Generation of 5-thio-2-nitrobenzoic acid was monitored as a function of time by recording the absorbance at 450 nm using a spectrophotometer.

### Incubation of homocysteine-thiolactone (HT) with low-density lipoprotein (LDL)

*In vitro *homocysteinylation of LDL was done according to the procedure of Vignini et al. [16]. Briefly, an aliquot of LDL (100 μg) was resuspended in 10 mM PBS (pH 8.2) and incubated with homocysteine-thiolactone (100 μmol/L, Sigma, St. Louis, MO) and the indicated amount (0.1 to 1.0 μg) of a total lipid fraction (TLb or TLp) with gentle stirring at 37°C for 2 h. After incubation, the mixture was passed through a Bio-gel P-2 column equilibrated with 10 mM PBS (pH 8.2) to remove any unreacted HT.

### Serum creatinine determination

The creatinine concentrations in rat serum samples were assayed by Jaffe's alkaline-picrate method as described by Adeoye *et al*. [29]. Proteins were precipitated from 1 ml of serum by the addition of 1 ml 10% sodium tungstate in 1 ml of 0.67 M sulfuric acid, and the supernatants were collected;1.5 ml of the supernatants were mixed with 0.5 ml of 2.5 M NaOH and 0.5 ml of 0.04 M picric acid, and then incubated for 5 min at 25°C. The absorbance at 520 nm was measured in each sample against a blank that consisted of distilled water with the reagent as described above using a spectrophotometer.

### Statistical analysis

Statistical analyses were performed for animal data using the GraphPad Prism 2.01 software package (GraphPad, San Diego, CA). Differences among diet groups with non-diabetic treatment or with diabetic treatment were analyzed using the one-way ANOVA (Table 2) test. Normalization of data distribution was given by Prism 2.01 before the comparison test. If the data were found to be parametric, they were analyzed by Tukey's multiple comparison test, followed by determination of differences vs. the control group after obtaining a significant ANOVA by the Dunnet's test. If the data were non-parametric, they were analyzed by the Kruskal-Wallis test of one way analysis of variance on rank, followed by Dunn's multiple comparison test. Differences among the diet treatment groups were considered significant if p < 0.01. For comparisons of size-frequency distributions, the difference of skewness was evaluated by the chi-square test for trend in the Prism 2.01 to evaluate the statistical difference of skewness. A value of *p *< 0.01 was considered a statistically significant difference.

**Table 2:** Final body weights, blood glucosse concentrations, and peripheral nerve or serum parameters in non-diabetic and diabetic rats (3 weeks) sacrificed at the end of the diet-feeding experiment

Treatment	Non-diabetic				Diabetic			
Diet	C	WR	BR	PR	C	WR	BR	PR
Group	C	WR	BR	PR	DC	DWR	DBR	DPR
Number of animals	4	6	6	6	4	9	9	9
Weight (g)	373.8 ± 7.8	374.7 ± 3.8	370.3 ± 9.1	384.8 ± 13.5	174.0 ± 8.6	174.2 ± 4.8	183.9 ± 2.7 ^b^	209.0 ± 3.3 ^a^*
Glucose (mg/dl)	127.0 ± 1.8	135.5 ± 2.7	129.0 ± 3.3	126.7 ± 4.0	438.0 ± 11.0	431.0 ± 9.3	405.2 ± 9.1	351.9 ± 6.3 ^a^*
NCV (m/s)	50.4 ± 0.8	49.9 ± 1.3	50.2 ± 0.8	50.4 ± 0.9	34.0 ± 1.4	34.4 ± 0.8	37.9 ± 1.1	44.5 ± 1.3 ^a^*
Na^+^/K^+^- ATPase (μmol/g/h)	6102.5 ± 218.9	4855 ± 463.4	6998 ± 515.6	6302 ± 371.5	2238 ± 102	2229.3 ± 153.0	2437.2 ± 189.9	3867.8 ± 177.6 ^a^*
Homocysteine (μM)	6.3 ± 0.3	5.9 ± 0.1	6.1 ± 0.2	6.3 ± 0.2	3.3 ± 0.2	3.1 ± 0.2	4.2 ± 0.1 ^b^*	5.3 ± 0.1 ^a^*
PON 1 (nmol/ml/min)	224.0 ± 11.1	217.9 ± 4.4	216.0 ± 4.6	239.5 ± 6.5	118.4 ± 7.4	117.4 ± 5.9	158.3 ± 8.4 ^a^	187.7 ± 13.6 ^a^*
HTase (nmol/mg/min)	9.8 ± 0.4	10.0 ± 0.2	10.2 ± 0.3	10.3 ± 0.3	2.0 ± 0.3 ^c^	1.8 ± 0.2 ^c^	4.3 ± 0.2 ^b^**	8.9 ± 0.2 ^a^**
Creatinine (μmol/l)	67.3 ± 1.7	60.3 ± 1.6	59.7 ± 1.3 ^a^*	59.5 ± 1.0 ^a^*	89.3 ± 1.7	88.3 ± 1.1 ^a^	82.5 ± 1.3 ^b^*	72.8 ± 1.1 ^c^*

One-way ANOVA was performed for 4 groups of non-diabetic treatment or diabetic treatment, followed by Tukey's multiple comparison test (if parametric). Difference vs. control (C group or DC group) was analyzed by Dunnet's multiple comparison test (*P < 0.01, **P,0.001). Kruskal-Wallis test was performed for the groups (if non-parametric) followed by Dunn's multiple comparison test. Values are means ± SEM. ^a,b,c^Values with different superscript letters are significantly different among the same column at P < 0.01.

In correlation analyses, Pearson correlation coefficients were calculated to identify potential associations of the HTase, PON1, NCV, and Na^+^/K^+^-ATPase activities. A value of *p *< 0.05 was considered a statistically significant difference. Data were presented as mean ± standard error of the mean (SEM).

## Results

### Body weight and blood-glucose level

The total mass of food consumed during the 3-week experimental diet period for each non-diabetic rat group was as follows: WR, 590 ± 31 g; BR, 611 ± 42 g; and PR, 631 ± 64 g. The total mass of food consumed during the same period for each diabetic rat group was as follows: DWR, 641 ± 58 g; DBR, 658 ± 30 g; and DPR 631 ± 53 g. The consumption rates were not significantly different among the normal and diabetic rats fed WR-, BR-, and PR-enriched diets (assessed by the Kruskal-Wallis test followed by Dunn's test).

The body-weight gain in the WR, BR, and PR groups was smooth, and no differences were observed among the three groups. Modest body-weight gain due to diabetes was observed in the DWR, DBR, and DPR groups, and considered to be a well-established phenomenon in insulin-deficiency caused by STZ treatment in the rat [30]. There were also no statistically significant differences in body-weight gain between rats fed a rice-enriched diet and those fed the control AIN93G diet. The DPR group, however, had statistically significant body-weight gain as compared with the DC group (*p *< 0.01, Dunnett's test) but not with the DBR group, as shown in Table 2.

All the blood-glucose levels of the non-diabetic AIN93G- and rice-diet groups (C, PR, BR and WR) were within the normal range during the experimental period (Table 2). In contrast, the blood-glucose levels of the diabetic rats fed the AIN93G, WR-, BR-, or PR-enriched diets (i.e., DC, DWR, DBR, and DPR groups, respectively) were elevated. During the initial week of the rice-enriched diet regimen, all diabetic groups demonstrated high blood-glucose levels (Table 2); after 3 weeks of the rice-enriched diets, however, the blood glucose levels of the DPR group were significantly lower than those of the DWR and DBR groups (*p *< 0.001, Tukey's test), as shown in Table 2.

### Amelioration of peripheral nerve dysfunction

**Figure 1:**
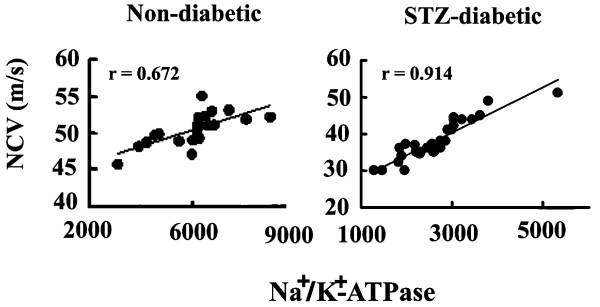
Correlation of NCV and sciatic-nerve membrane Na^+^/K^+^-ATPase activities in non-diabetic rats (n = 22) and STZ-induced diabetic rats (n = 31). Non-diabetic rats consisted of C-group (n = 4), WR-group (n = 6), BR-group (n = 6), and PR-group (n = 6). STZ-diabetic rats consisted of DC-group (n = 4), DWR-group (n = 9), DBR-group (n = 9), and DPR-group (n = 9). Regression lines are shown for analyses that include all data points (n = 22 and n = 31). Pearson's correlation coefficients (r) were used to evaluate simple linear relationship between variables.

A positive correlation was established between values for NCV and Na^+^/K^+^-ATPase activities in non-diabetic and STZ-diabetic rats (Fig. 1). The data also include rats fed by a regular diet (AIN93G). This result demonstrates that decreased sciatic-nerve Na^+^/K^+^-ATPase activities are likely to result in electrophysiological and neurophysiological abnormalities. Comparison of the effects of the diet intakes on NCV and Na^+^/K^+^-ATPase activity is shown in Table 2, expressed as mean ± SEM. Diabetic rats fed the control diet (AIN93G) showed significantly decreased NCVs when compared with those in the non-diabetic rats. NCVs in the DPR group, on the other hand, were significantly faster than those observed in the DC, DWR or DBR group (*p *< 0.001, Tukey's test). Na^+^/K^+^-ATPase activities in the DPR group were also significantly higher than those seen in the DBR, DWR, and DC groups (*p *< 0.001, Tukey's test), as shown in Table 2.

### Morphometric analysis of sciatic nerve

Size-frequency distribution histograms for myelinated fiber diameter, myelinated axon diameter, and the G ratio in rat sciatic nerves were evaluated by the *chi*-square test for trend in the Prism 2.01, and compared among three groups: a non-diabetic group fed the AIN93G diet (C group); a diabetic group fed the AIN93G diet (DC group); and a diabetic group fed the PR diet (DPR group). There was no statistically significant difference between myelinated fiber distributions; one difference between axonal distributions for the C and DC groups, however, indicated that the STZ rat model of diabetes primarily affected axons. Since the G ratio is a reflection of the diameters of axons and myelinated fibers, these observations indicate that PR intake could prevent or correct myelinated axonal injury in the STZ model of diabetic neuropathy (data not shown).

### PON1 and HTase

Serum activities for creatinine or PON1 and HTase in the diabetic and non-diabetic animals are shown in Table 2 as means ± SEM. Given that the non-diabetic groups had similar weight gains and diet consumptions, we cannot attribute the changes in serum homocysteine concentrations to differences in vitamin or amino-acid intake in the rice diets. The decreased homocysteine levels in the diabetic rats indicate that increased creatinine levels may reflect diabetic complications such as nephropathy. The serum homocysteine level and PON1 activity in the DPR group sera were affected by STZ-induced diabetes, which was not the case in the DC, DWR and DBR groups (*p *< 0.001, Tukey's test) (Table 2).

To determine whether the PR-diet promoted amelioration of the nervous function by a specific active component of PR due to an HT-related mechanism, a correlation analysis was performed between activities of Na^+^/K^+^-ATPase and HTase (or PON1) for each group of Table 2. A positive correlation was established between activities of serum HTase and PON1 in all the diet-treated and the control groups (r = 0.819 to 0.992). On the other hand, a positive correlation between Na^+^/K^+^-ATPase and HTase activities was established only in the DPR (r = 0.913, p < 0.001) and PR groups (r = 0.878, p < 0.05). Other diet-treated groups showed no correlation between the two enzyme activities. Although the DBR group showed an improvement in diabetic neuropathy, there was no correlation between the two enzyme activities in DBR group (r = -0.217, non-significant) and BR group (r = -0.142, non-significant).

### Homcysteine-thiolactonation of LDL

**Figure 2:**
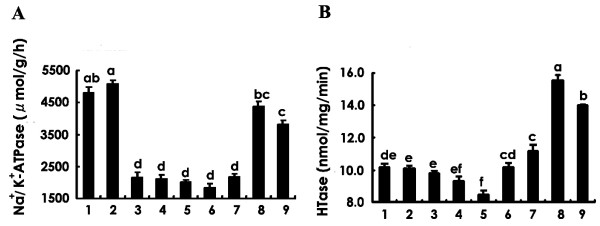
(A) Effects of modified LDL on Na^+^/K^+^-ATPase activity. Activity was assayed by incubation with the following: 1, no additive; 2, LDL; 3, HT-treated LDL ; 4, TLb (0.1 μg)/HT-treated LDL; 5, TLb (1.0 μg)/HT-treated LDL; 6, TLb (10 μg)/HT-treated LDL; 7, TLp (0.1 μg)/HT-treated LDL; 8, TLp (1.0 μg)/HT-treated LDL ; 9, TLp (10 μg)/HT-treated LDL. (B) Effects of total lipid extract on HTase activity. Activity was assayed by incubation with the following: 1, no additive; 2, TLb (0.1 μg); 3, TLb (0.5 μg); 4, TLb (1.0 μg); 5, TLb (5.0 μg) ; 6, TLp (0.1 μg); 7, TLp (0.5 μg); 8, TLp (1.0 μg); 9, TLp (5.0 μg). Values are means ± SEM, n = 6 individual experiments. The data values were analyzed by one-way ANOVA followed by Tukey's multiple comparison test. ^a,b,c,d,d,f^Values with different superscript letters differ significantly among the columns at *p *< 0.05.

HT causes LDL deterioration, resulting in the development of diabetic complications as a vascular risk factor by HT-modified LDL. HTase is present in serum HDL and has an important role in the antioxidation of LDL. We hypothesized that the efficacy of a PR diet on diabetic neuropathy might be dependent on an HT-related mechanism. To test this possibility, an additional effect of rice bran lipid extracted fractions (TLp or TLb) on Na^+^/K^+^-ATPase activity was tested with HT-treated LDL. Changes in the Na^+^/K^+^-ATPase activity of normal rat sciatic nerve was studied by incubation of HT-treated LDL with HT and TLp or TLb (Fig. 2A). A significant decrease in Na^+^/K^+^-ATPase activity occurred in an *in vitro *system incubated with HT-modified LDL as compared with intact LDL. Incubation with TLp (0.1 μg to 10 μg) attenuated the inhibitory activity of HT-modified LDL on Na^+^/K^+^-ATPase activity (columns 7 to 9, Fig. 2A), whereas TLb had no effect on the inhibition of Na^+^/K^+^-ATPase activity by HT-modified LDL (columns 4 to 6, Fig. 2A). Neither TLp nor TLb alone produced any modification of Na^+^/K^+^-ATPase activity (data not shown). The effect of TLp suggests the presence of an inhibitory factor causing normalization of Na^+^/K^+^-ATPase activity that has been blocked by HT-modified LDL.

### HTase activity of HDL

In the previous experiment, we showed that Na^+^/K^+^-ATPase activity was controlled by HT-modified LDL. The efficacy of PBR on diabetic neuropathy may be related to the HTase activity of HDL on an HT-related mechanism. To verify this possibility, the effect of rice bran lipid extracted fractions (TLp or TLb) on HTase activity was tested. HDL prepared from normal male Wistar rat serum was used as an enzyme source of HTase as described in the Methods section. HTase activity in the presence of TLp or TLb (0.1 to 5.0 μg) was compared. TLp treatment showed a statistically significant difference HTase activity as compared with treatment with TLb (Fig. 2B). TLp had a dose-dependent stimulatory effect (0.1 μg to 1.0 μg) on HTase activity (columns 6 to 8 in Fig. 2B), and more than 5.0 μg produced saturation of this effect. In contrast, TLb had no stimulating effect on HTase activity in the same dosage range (columns 2 to 4, Fig. 2B).

**Figure 3:**
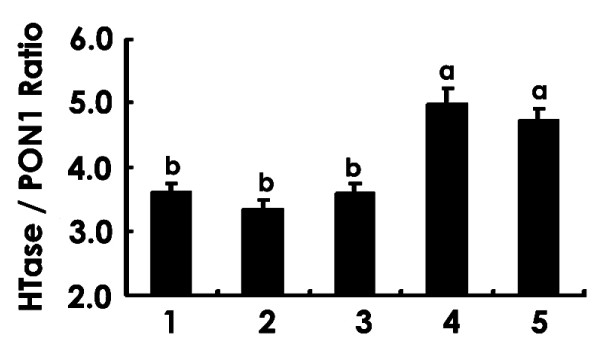
Effects of TLp on the ratio of HTase/PON1 activities. HTase and PON 1 activities of HDL were assayed by incubation with the following: 1, no additive; 2, TLp (0.1 μg); 3, TLp (0.5 μg); 4, TLp (1.0 μg); 5, TLp (5.0 μg). HTase activity corresponds to combined enzyme activities of PON1 and PON3. Values are mean ± SEM in individual experiments (n = 6). The data were analyzed by one-way ANOVA followed by Tukey's multiple comparison test. ^a,b^Values with different superscript letters differ significantly among the columns at *p *< 0.05.

On the other hand, PON1 activity in HDL was assayed using paraoxyethyl as a substrate, and there was no change upon the addition of TLp in the above dosage range (data not shown). This finding suggests that other PON isozymes may be responsible for TLp-induced HTase activation. The HTase/PON1 activity ratio calculated for each HDL sample showed a dose-dependent elevation by TLp (Fig. 3). One- and 5 μg of TLp gave a statistically significant ratio increase as compared with lower amounts of TLp. This suggests that TLp has no effect on the activities of PON1, whereas it does have an effect on an isozyme such as PON3.

## Discussion

In previous studies, a PR diet was reported to lower the elevation of blood glucose, type-1 plasminogen activator inhibitor, and lipid peroxide concentrations in STZ-induced diabetic rats [2,3]. This finding suggested that PR intake can ameliorate diabetic vascular complications, such as myocardial infarction, retinopathy, and nephropathy.

We evaluated the effects of a PR diet on diabetic complications, especially peripheral neuropathy, in STZ-induced diabetic rats. We found that PR and BR diets lowered blood-glucose concentrations in diabetic rats, and that the PR diet had a statistically significant blood-glucose lowering effect (*p *< 0.01) as compared with the blood-glucose levels measured in rats on the BR diet (Table 2).

Although the non-diabetic and STZ-diabetic groups ate the same amount of diet, there was different gain in body weight. As the total energy available from protein, fat, and carbohydrate did not differ among the 3 diets (Table 1), the same intake of diet suggests that STZ-diabetic rats cannot sufficiently utilize the energy available from their diet. We speculate that the STZ-treatment injured the insulin-secreting beta cells, impairing glucose uptake into skeletal muscle and resulting in weight loss. Alternatively, the effect of STZ on hormonal functions of the adrenal glands might enhanced the basal metabolic rate. In the STZ-diabetic rats, the concentrations of noradrenaline are known to increase in a various tissues in these animals [31,32]. Thermogenesis is under control of the sympathetic nervous system, as noradrenaline has the ability to control biochemical pathways that increases mitochondrial oxidation rates and ATP synthesis, hence leading to an increase in heat production and not leading to the gain in body weight [33].

Our experiments showed that diabetic neuropathy was induced by STZ-treatment at 5 weeks, as evidenced by slowing of NCV and impairing of Na^+^/K^+^-ATPase activity (Table 2), as well as to pathological changes consisting mainly of axonal degeneration in the sciatic nerves of the diabetic rats (data not shown). NCV values for all of the experimental rats, including diabetic and non-diabetic ones, correlated well with the sciatic-nerve membrane Na^+^/K^+^-ATPase activity values of individual diabetic and non-diabetic rats (Fig. 1). This result substantiated that measurement of Na^+^/K^+^-ATPase activity provides an additional measure to NCV for evaluating the severity of peripheral-nerve dysfunctions in animals. In our current study, the efficacy of the PR diet also was confirmed by morphometric analysis showing that axonopathy predominates over myelinopathy in STZ-diabetic rats. Abnormal fibers undergoing axonal degenerative changes and myelin breakdown are reported to appear in the sciatic [34] and phrenic [35] nerves of STZ-induced diabetic rats during prolonged hypoglycemia. Skewing of the G ratio was corrected by the PR diet, giving an axonal diameter distribution similar to that for the non-diabetic animals.

Serum Hcy is a known risk factor for vascular disease and neurodegenerative disease [36] because an excess amount of Hcy generates HT with high toxicity [37]. Abnormal serum Hcy concentrations also have been observed in diabetic patients who develop peripheral vascular diseases. In our study, the total serum homocysteine of the DC group showed a decrease to 52.4% of the level for the C group (Table 2), a finding in agreement with those of previous studies [38-40]. The increase in serum creatinine in the DC group was probably due to complications caused by renal failure. The decrease in serum Hcy may have been produced by abnormal renal clearance. There is a possibility that in the beginning stages of STZ-induced diabetes, hepatic trans-sulfuration and re-methylation of the enzymes cystathionine β-synthase and cystathionine γ-lyase may increase in STZ-induced diabetic animals due to lack of insulin [40].

Although in our study the Hcy level decreased in the DC group, PR intake normalized the serum Hcy level as compared with that seen in WR- or BR-intake. Serum PON 1 activity decreases in STZ-induced diabetic rats [20] and in patients with vascular complications caused by type 2 diabetes mellitus [21,41-43]. PON has 3 isozymes, PON1, PON2, and PON3, which have distinct substrate specificities and tissue localization [17]. PON1 is associated with HDL and has been shown to reduce LDL susceptibility to lipid peroxidation. PON2 does not appear in serum. Whether the total HTase activity in human sera are affected by PON3 activity has not yet been determined [44]. PON1 is the only enzyme capable of hydrolyzing paraoxon, a metabolite of organophosphate parathion. PON1, PON2, and PON3, however, have the ability to hydrolyze a number of acyl-homocysteine thiolactone molecular species [17]. Serum Hcy produces HT that is toxic to the vascular system in diabetes. The activity of PON1 in serum and that of HTase in HDL decreased in STZ-induced diabetic rats, as reported previously [20,21]. Diabetic rats on a PR diet showed significant attenuation of PON1 and HTase activities as compared with those on a control diet. There is a significant correlation of both enzyme activities in diabetic rats on PR and BR diets. Interestingly, there also is a highly significant correlation between HTase and Na^+^/K^+^-ATPase activities of diabetic rats on a PR, but not a BR, diet. The significant association between these two enzymes may reflect the greater effectiveness of PR than of BR intake.

To investigate whether PR has an active factor(s) making it more effective than BR, Na^+^/K^+^-ATPase activity was examined after incubation with HT-treated LDL and total lipids extracted from PR and BR brans (TLp and TLb, respectively). We found a significant decrease in Na^+^/K^+^-ATPase activity on incubation with HT-modified LDL as compared with incubation with intact LDL (*p *< 0.05) (Fig. 2A). Hcy has been reported to produce oxidative stress in rat hippocampal neurons by inhibiting Na^+^/K^+^-ATPase activity [14,15]. Vignini et al. [16] reported that HT-modified LDL attenuates Na^+^/K^+^-ATPase activity in cultured human aortic endothelial cells. We obtained a similar finding for the peripheral nervous system of diabetic rats. In our experiment, TLp (0.1 to 10 μg)/HT-modified LDL gave a weak inhibition of Na^+^/K^+^-ATPase activity, whereas TLb/HT-modified LDL gave the same inhibition of Na^+^/K^+^-ATPase activity as did HT-modified LDL. Neither TLp nor TLb alone produced any modification of Na^+^/K^+^-ATPase activity (data not shown). This finding may suggest that TLp bears an inhibitory factor(s) that causes deprivation or attenuation of the effect of HT-treated LDL on Na^+^/K^+^-ATPase activity. This finding also may help to clarify the mechanism of the amelioration of diabetic neuropathy by PR intake. We demonstrated that TLp also directly enhanced the HTase activity of HDL (Fig. 2B). To estimate the enzyme activity ratio of PON3 to PON1+PON3, we assayed each enzyme activity by a substrate specificity of PON1 and HTase. The enzyme activity ratio of PON1 to HTase (Fig. 3) suggests that this enhancement may be due to the PON 3 present in HDL. PON3 is an HDL-related glycoprotein with multi-enzymatic properties and antioxidant activity, considered to aid in preventing LDL oxidation. PON3 bears many conserved phosphorylation and N-glycosylation consensus sites [45]. Modification at any of the sites required for activity *in vivo*, however, has yet to be shown. Possibly, PON3 protein has a hydrophobic binding site(s) against a lipid that regulates its activity. It is also possible that a certain unknown lipid component in PR may bind to PON3 and activate it. PR is expected to lower the postprandial blood-glucose increase, as is BR as compared with WR. Our research suggests that this efficacy may be based on higher concentrations of dietary fiber in BR and PR as compared with the fiber in WR. The PR diet includes almost the same amount of dietary fiber as BR. Our study showed, however, that the PR diet lowered rat blood-glucose levels significantly more than did BR (Table 2). Thus, compared with the BR diet, except for lowering of the blood-glucose level, which may be due to another PR intake mechanism, the PR diet mitigates diabetic neuropathy and is much more effective in easing peripheral nerve dysfunction.

## Conclusion

We found that PR-diet intake in STZ-induced diabetic rats provided protection against deterioration of the peripheral nerve. An HTase-related mechanism contributed to the improvement of diabetic neuropathy. The total lipid extract of PR protected Na^+^/K^+^-ATPase from toxic HT-modified LDL and enhanced HTase activity of HDL. This protection could contribute to the efficacy of the PR diet in ameliorating the deleterious effects of diabetic neuropathy

## Abbreviations

The abbreviations used are: 

C: AIN93G diet control; 

G ratio: Axonal diameter to total fiber diameter ratio; 

Hcy: Homocysteine; 

HTase: Homocysteine-thiolactonase; 

HT: Homocysteine-thiolactone; 

PBS: Phosphate-buffered saline; 

PON: Paraoxonase; 

STZ: Streptozotocin; 

LDL: Low-density lipoprotein; 

HDL: High-density lipoprotein; 

ANOVA: Analysis of variance.

## Competing interests

The author(s) declare that they have no competing interests.

## Authors' contributions

SU participated in the concept and design of the study, data collection, tissue sampling, statistical analysis and drafting the manuscript.

YI participated in animal experimentation.

KM participated in animal experimentation and carried out nutrients analysis

MK participated in the design of the animal study.

TA participated in lipid isolation and carried out lipid analysis.

MR participated in electrophysiology.

RKY participated in the design and interpretation of the study and drafting of the manuscript.
